# A case report and literature review on TP53 gene mutation in a bladder rhabdomyosarcoma patient

**DOI:** 10.3389/fonc.2024.1396368

**Published:** 2024-07-30

**Authors:** Yixuan Li, Tianci Yang, Zhouhang Zheng, Zhong Wang, Shahzard Muhammad Umar, Ning Fan, Shenbao He, Wei Chang, Wei Wang

**Affiliations:** ^1^ The Second Clinical Medical School, Lanzhou University, Lanzhou, China; ^2^ The Clinical Medical School, Yan’an University, Yan’an, China; ^3^ Department of Urology, The Second Hospital of Lanzhou University, Lanzhou, China

**Keywords:** bladder tumor, rhabdomyosarcoma, TP53, gene mutation, case report

## Abstract

**Background:**

Rhabdomyosarcoma of the bladder is an infrequent neoplastic condition characterized by a pronounced malignant situation with challenges in treatment due to the lack of standardized guidelines and large-scale of clinical studies. The patient in this case is tested TP53 mutation that may provide new diagnostic and therapeutic options.

**Case presentation:**

Here, we reported a 34-year-old male who received bladder tumor resection, and diagnosed as bladder rhabdomyosarcoma with TP53 mutation after the pathology test. This patient underwent 6 rounds of chemotherapy. However, the pelvic tumor recurred 11 months after the first surgery. So, the patient accepted the pelvic tumor resection. Only 3 months after the surgical intervention, the patient underwent abdominal massive metastasis and ultimately succumbed to the illness six months following the second surgery. The course of the illness was 22 months.

**Conclusion:**

Bladder rhabdomyosarcoma is a disease with an extremely poor prognosis. Genetic testing holds significant value in the diagnosis and treatment. Perhaps targeted therapy against TP53 is potential valuable for such rare diseases.

## 1 Introduction

Rhabdomyosarcoma (RMS) is the most common soft-tissue sarcoma which arises in primitive fetal mesenchyme cells. It is usually happened in the first two decades of life (1) and rarely occurs in the genitourinary tract (2). Bladder rhabdomyosarcoma is a rare malignant tumor originating from mesodermal stromal tissue, with a mixed histology of bladder embryonal and spindle cells being uncommon. TP53 is a kind of tumor suppressor gene and its mutation is one of the most frequent alterations in human cancers (3). It is reported that RMS is associated with a high rate of TP53 mutation (4, 5). A single patient diagnosed as a mixed rhabdomyosarcoma consisting of bladder embryo and spindle cells was admitted to our hospital, January in 2022. Due to the patient is combined with the mutation of TP53, we detail the course of the patient’s disease and treatment as well as his prognosis and discuss possible treatments to target the mutations. The case is detailed as follows.

## 2 Case presentation

**Figure 1:**
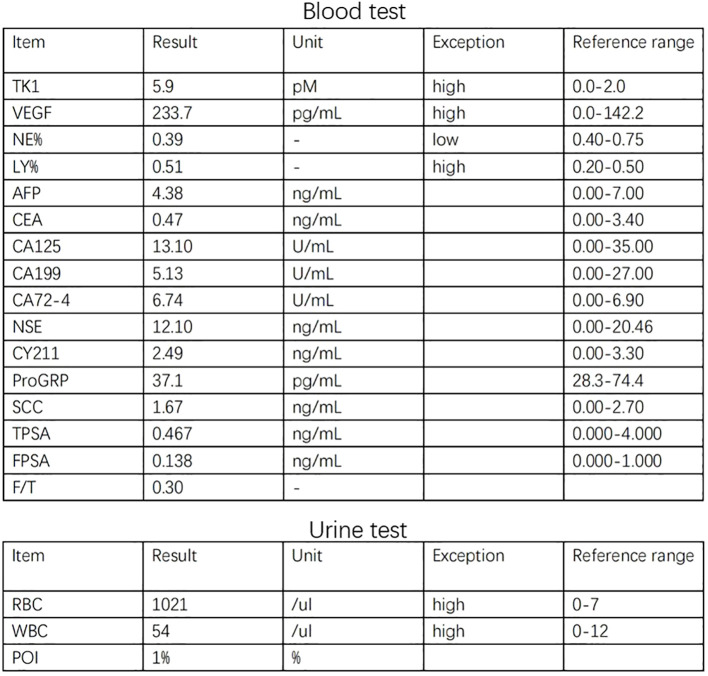
The graph shows higher expression of TK1 and VEGF in blood and higher WBC and RBC in urine.

**Figure 2:**
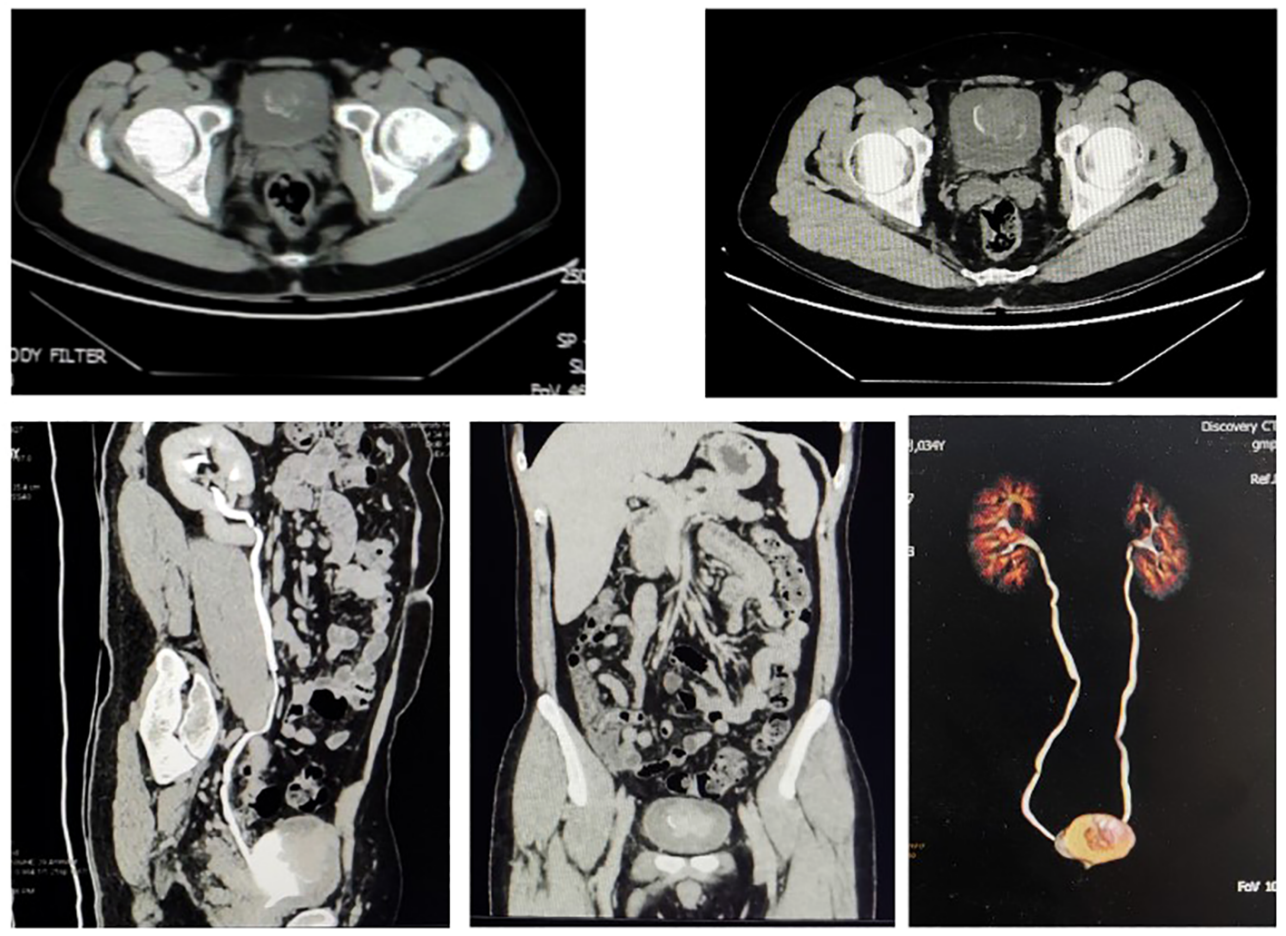
CT scan of the abdomen with contrast reveals the bladder tumor.

The 34-year-old male patient presented with some concerns about intermittent painless gross hematuria for about a week. The hematuria occurred obviously, which is intermittent and more pronounced at night. The patient had no family history of genetic predisposition to this condition. A urological ultrasound conducted at an external medical facility indicated suspicion of a bladder tumor. Upon admission, the patient’s physical examination revealed a body temperature of 36.2°C, a heart rate of 86 beats per minute, a blood pressure of 125/87mmHg, and a weight of 65kg, with stable vital signs. During the hospitalization, a routine blood test showed that serum tumor markers were at normal levels.TK1 and VEGF are higher than normal levels, which indicates poor prognosis (**Figure 1**). The urine test showed that RBC and WBC were high. Specialist examination revealed no bulges in the renal areas, no percussion pain, no tender points along the bilateral ureteral walking area, and no bulges or tenderness in the suprapubic bladder region. Subsequently, an abdominal CT scan was performed, which revealed a thickened bladder wall, a cauliflower-like mixed density occupancy on the anterior wall of the bladder measuring approximately 3.7*3.9cm, visible calcifications at the edges, and moderate enhancement of the lesions upon contrast-enhanced scanning. The umbilical ureter is affected by a lesion with some upward extensional changes, and no definite enlarged lymph nodes were observed retroperitoneally. There were no apparent abnormalities in the remaining abdominal organs. (**Figure 2**) Given these findings, the potential presence of a malignant bladder tumor was considered. Consequently, the patient was admitted to The Second Hospital of Lanzhou University for further diagnosis and treatment. A laparoscopic partial cystectomy was performed, revealing a spherical tumor measuring 4cm × 4cm on the anterior wall of the bladder’s apex, which did not invade the outer tissue of the bladder wall. The tumor and the 1cm area of the bladder wall were excised, and the specimens were sent for pathological examination.

## 3 Results

### 3.1 Blood and urine test result

(**Figure 1**) The graph shows higher expression of TK1 and VEGF in blood and higher WBC and RBC in urine.

### 3.2 Preoperative CT image

(**Figure 2**) CT scan of the abdomen with contrast reveals the bladder tumor.

### 3.3 Pathological results

**Figure 3:**
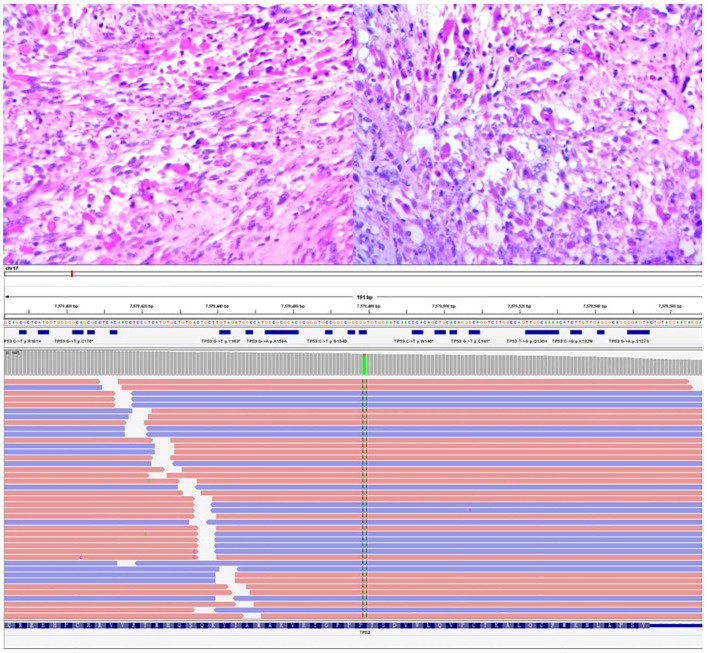
The image depicted above displays the histopathological section of a tumor under a 400x magnification microscope, following hematoxylin and eosin staining, and mutation fragment of TP53 gene.

**Figure 4:**
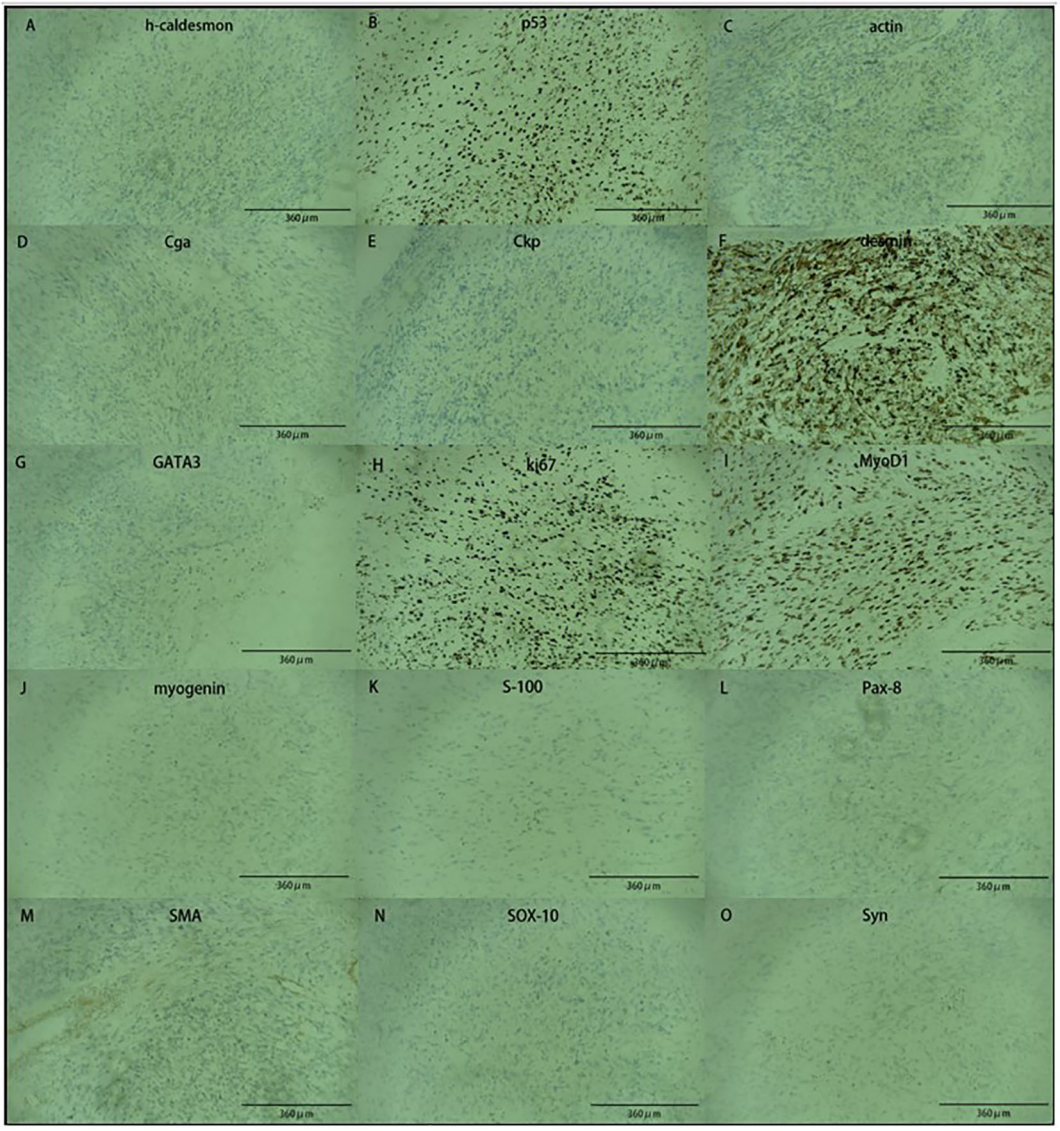
The image showed immunohistochemical results. Tumor cells are positive for **(B, C, F, H, I)**, and negative for **(A, D, E, G, J, K, L, M, N, O)**.

The bladder tumor was identified as rhabdomyosarcoma (RMS), specifically of the embryonic and spindle cell mixed type, based on pathological findings (**Figure 3**). Immunohistochemical analysis revealed the following results: Tumor cells were negative for Ckp, positive for desmin and actin, negative for myogenin, positive for MyoD1, and negative for CgA, S-100, Syn, h-caldesmon, SMA, Sox-10, Pax-8, and GATA3. Mutant expression of P53 was observed. PD-L1 expression is negative and 80% of the tumor cells were positive for Ki67 (**Figure 4**). Genetic testing and examination revealed that TP53 p.P151S; MET amplification; The abundance of TP53 mutations was 88.1%.

### 3.4 Genetic variation results

TP53 mutation, exon5c.451C>T pP151S (**Figure 3**).

(On exon 5, cytosine C at position 451 is mutated to thymine T, and its complementary chain is shown as adenine A, resulting in the change of proline P to serine S at position 151).

### 3.5 CT image of tumor metastasis after the second surgery

**Figure 5:**
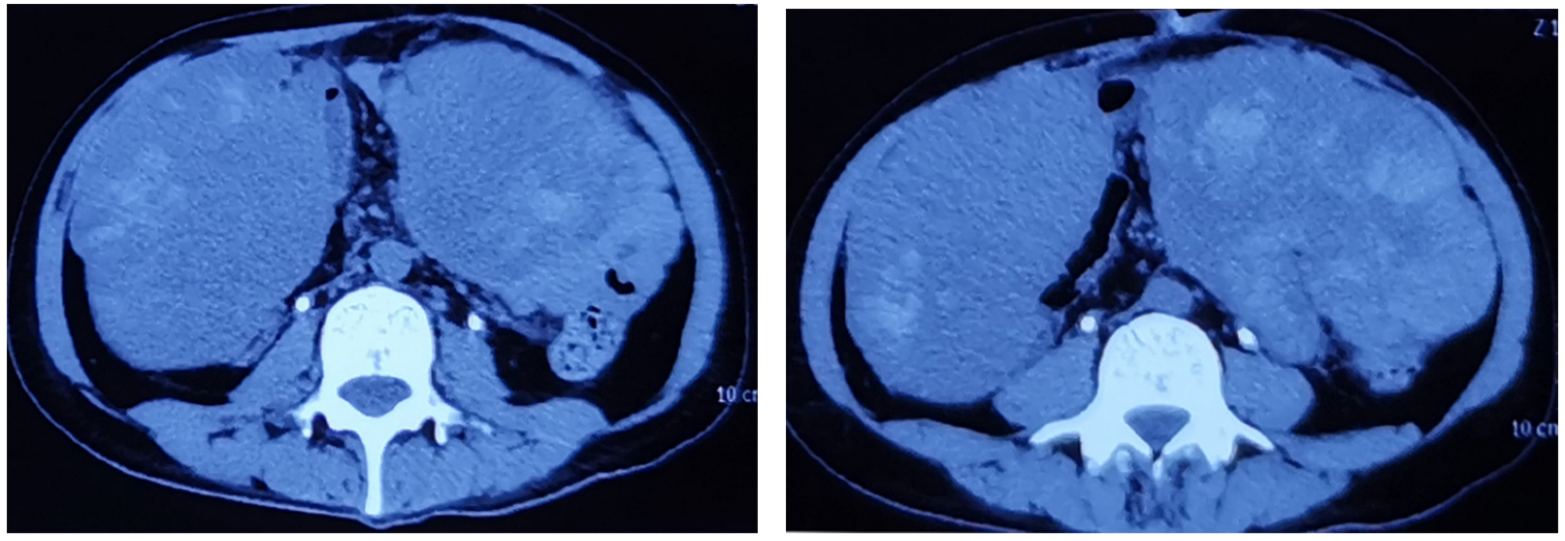
CT scan after the second operation showed extensive metastasis of the abdominal cavity. There is a tumor of 15*12cm on the left and an 18*13cm tumor on the right.

(**Figure 5**) CT scan after the second operation showed extensive metastasis of the abdominal cavity. There is a tumor of 15*12cm on the left and an 18*13cm tumor on the right.

## 4 Treatment

In this case, the PD-L1 protein was assessed and found to have negative expression. In treatment, the consensus among experts suggests a combination of surgery, VAC therapy, and radiotherapy. The patient rejected radiotherapy. Genetic testing data was consulted, leading to the decision to employ laparoscopic partial cystectomy and VAC chemotherapy to preserve the bladder. Following surgery, the patient underwent six rounds of chemotherapy. Despite these efforts, the tumor reappeared in 11 months after surgery, the size reached up to 8cm*8cm. The patient received pelvic tumor resection in another hospital. However, CT showed the tumor has metastasized to the abdominal cavity 3 months after the surgery. A huge tumor was inside the abdominal cavity, 15*12cm on the left side and 18*13cm on the right. Regrettably, the patient passed away six months after the surgical procedure.

## 5 Discussion

Rhabdomyosarcoma is a malignant tumor composed of rhabdomyoblasts at different stages of differentiation, which is more likely to occur in childhood and adolescence, with two peaks of 2~6 years old and 15~19 years old, rarely occurring in adulthood, slightly more in males than females (6); Genitourinary rhabdomyosarcomas occur in less than 20 percent of RMS patients (7).

Rhabdomyosarcoma (RMS) is categorized into two subtypes, embryonal rhabdomyosarcoma (ERMS) and alveolar rhabdomyosarcoma (ARMS), based on the cellular characteristics observed under light microscopy (ARMS) (8), ERMS and ARMS are additionally categorized based on histological or molecular features, and can be further classified as either alveolar or embryonal RMS. These subtypes include grape-like RMS and spindle/sclerosis RMS (9). RMS has the potential to develop in various anatomical locations, with urinary rhabdomyosarcoma commonly manifesting in the bladder triangle and bladder neck. Though specific symptoms of bladder rhabdomyosarcoma are not well-defined, as the tumor grows and obstructs, it can lead to urinary tract obstruction, increased urinary frequency, urgency, painful urination, abdominal discomfort, and presence of blood in the urine (6). The etiology of RMS is not fully understood, but it is believed to be influenced by genetic factors and environmental factors (10). According to the staging criteria established by the Children’s Oncology Group (COG), the initial diagnosis of this case was T1N0M0, clinical stage II, with postoperative pathological group I and low-risk stratification. Rhabdomyosarcoma is a highly aggressive tumor with a poor prognosis, posing significant challenges for treatment. The primary approach involves surgical intervention, complemented by chemotherapy and radiotherapy (11).

Following laparoscopic removal of a bladder tumor, the histopathological analysis revealed the presence of embryonic and spindle-cell mixed rhabdomyosarcoma in the patient. Inquiries were made regarding the patient’s familial and genetic background, and it was determined that neither the patient’s immediate nor extended family had a history of other systemic tumors. Furthermore, based on established diagnostic criteria, conditions such as Li-Fraumeni syndrome, bladder urothelial carcinoma, pheochromocytoma, and bladder neurofibroma were ruled out. However, it was found that the patient harbored a mutation in the TP53 gene.

The TP53 gene is situated on chromosome 17 and functions as a tumor suppressor gene. Disruption of the TP53 pathway can greatly expedite the progression of tumors (12). Patients with RMS have mutations in the TP53 gene, but the mutation rate is not high. In a study of 135 children with RMS, only 18 of then developed TP53 mutations. The study showed that the prevalence of RMS with mutations varied widely by pooling cases of pediatric patients with RMS who had been tested for TP53 in different regions of Europe and the United States, and analyzing the mutation frequency and mutant exons separately (8). In addition, a study of both pediatric and adult patients with rhabdomyosarcoma showed similar prevalence (3 out of 18 patients) and a worse prognosis for RMS with TP53 mutations (13). Therefore, testing TP53 gene mutations in young cases facilitates early detection, treatment, and improved patient survival rates.

Mutation in TP53 gene is widely seen, approximately to 50% in different kinds of human cancers (14, 15). Mutant TP53 proteins have been proposed to drive malignant transformation and sustain tumor growth through multiple processes (16). It is reported that there are two potential approaches to TP53-targeted therapy. One hypothesis shows that siRNA- or shRNA-mediated knockdown of mutant TP53 genes, the other shows to restore the function of wild type TP53 in tumor cells expressing mutant TP53. Both may become promising strategies to suppress tumor expansion (17). In addition to the therapeutic options provided by the guidelines, targeted therapy against the TP53 gene offers new treatment for patients with rhabdomyosarcoma combined with TP53 mutations.

## 6 Conclusion

In summary, we reported a case of bladder rhabdomyosarcoma with a mutation of gene TP53. Based on the patient’s blood test results, as well as pathology and immunohistochemistry, we have predicted that the patient’s prognosis was poor. As rhabdomyosarcoma has a high degree of malignancy, and there is currently no other recommended regimen, the patient received regular therapy of bladder cancer, including surgery and chemotherapy. The fact revealed that the disease is progressing rapidly. It remains to be proved whether this is related to the mutation of TP53. Nevertheless, this case provides new ideas for exploring treatment for rhabdomyosarcoma combined with TP53 mutations. Genetic testing serves as a valuable adjunctive diagnostic tool, offering pertinent data for the identification of appropriate chemotherapeutic agents and targeted therapy for rare cases. Genetic testing may also help predict prognosis. The treatment against target gene methods still needs to be further explored and studied.

## Data availability statement

The original contributions presented in the study are included in the article/supplementary material. Further inquiries can be directed to the corresponding author.

## Ethics statement

Written informed consent was obtained from the individual(s) for the publication of any potentially identifiable images or data included in this article.

## Author contributions

YL: Writing – original draft, Writing – review & editing. TY: Investigation, Writing – review & editing. ZZ: Investigation, Writing – review & editing. ZW: Investigation, Writing – review & editing. SU: Software, Writing – review & editing. NF: Software, Writing – review & editing. SH: Software, Writing – review & editing. WC: Software, Writing – review & editing. WW: Conceptualization, Funding acquisition, Project administration, Resources, Supervision, Validation, Writing – review & editing.
